# Does propofol definitely improve postoperative cognitive dysfunction?—a review of propofol-related cognitive impairment: Propofol-related cognitive impairment

**DOI:** 10.3724/abbs.2022067

**Published:** 2022-06-09

**Authors:** Pengfei Liu, Sheng Zhao, Hui Qiao, Tianzuo Li, Weidong Mi, Zhipeng Xu, Xinying Xue

**Affiliations:** 1 Department of Anesthesiology Beijing Shijitan Hospital Capital Medical University Beijing 100038 China; 2 Department of Cardiology Fuwai Hospital National Center for Cardiovascular Disease Chinese Academy of Medical Science and Peking Union Medical College Beijing 100037 China; 3 Anesthesia and Operation Center the First Medical Center Chinese PLA General Hospital Beijing 100853 China; 4 Department of Respiratory and Critical Care Beijing Shijitan Hospital Capital Medical University Beijing100038 China

**Keywords:** postoperative cognitive dysfunction, propofol, inflammation, neurotransmitter, synaptic plasticity, non-coding RNA

## Abstract

Postoperative cognitive dysfunction (POCD) is a common brain function-related complication after surgery. In addition to old age being an independent risk factor, anesthetics are also important predisposing factors. Among them, propofol is the most commonly used intravenous anesthetic in clinical practice. It has a rapid onset, short half-life, and high recovery quality. Many studies report that propofol can attenuate surgery-induced cognitive impairment, however, some other studies reveal that propofol also induces cognitive dysfunction. Therefore, this review summarizes the effects of propofol on the cognition, and discusses possible related mechanisms, which aims to provide some evidence for the follow-up studies.

## Introduction

Postoperative cognitive dysfunction (POCD) usually appears after surgery, and its incidence increases in the elderly (> 65 years). POCD is a complication characterized by impaired memory, decreased information processing ability, and decreased attention, and it is accompanied by a series of emotional changes [1]. POCD not only leads to a decline of quality of life but also heralds further deterioration. A previous study in 2008 showed that the 1-year mortality rate of patients with POCD within 3 months after surgery is almost twice of patients without POCD [2]. Among them, age was an independent risk factor for POCD. According to the International Postoperative Cognitive Dysfunction Research data, the incidence of POCD in elderly patients within 7 days and 3 months after surgery is 25.8% and 10%, respectively [3]. The long-term existence of POCD significantly increases the risk of postoperative death in patients [1].

In addition to age as an independent risk factor for POCD, anesthetics can also affect cognitive function. In recent years, an increasing number of studies have reported the effects of anesthetics on cognitive dysfunction [ 4, 5] . In these studies, propofol, a commonly used intravenous anesthetic during the perioperative period, is known to have a rapid onset, few adverse reactions, and no significant accumulation of continuous infusion, and is widely used in clinical practice. Propofol (2,6-isopropylphenol) can bind to the β subunit of the γ-aminobutyric acid receptor to change the chloride channel. Propofol can also simultaneously inhibit the release of acetylcholine and glutamate, resulting in sedative and hypnotic effects [6]. At present, a large number of basic and clinical studies have explored the protective effects of propofol on cognitive impairment [ 7, 8] . Propofol can inhibit oxidative stress and inflammation, stabilize calcium overload, reduce endoplasmic reticulum stress, improve mitochondrial energy metabolism, and play a protective role in cognitive dysfunction induced by surgical trauma and ischemia reperfusion [ 9, 10] . However, propofol anesthesia has been reported to cause cognitive impairment [ 11, 12] .

Therefore, this review summarizes the role of propofol in cognitive impairment and its potential mechanisms, which would be of great significance for guiding the clinical application of propofol.

## Propofol Anesthesia and POCD in Clinic Studies

Elderly patients have decreased learning and memory abilities and weakened brain function, therefore, postoperative cognitive dysfunction is more common. Many studies compared the effects of propofol anesthesia and inhalation anesthesia on the POCD in the elderly patients [ 13– 16] . However, the results are still in dispute. Several studies reported that propofol anesthesia could reduce the incidence of POCD and found that propofol anethesia could improve cognitive function of the patients undergoing thoracic surgery [ 13, 14] . Ding *et al*. [15] also reported the neuro-protective effects of propofol anethesia, rather than sevoflurane inhalation in patients with abdominal surgery. Zhang *et al*. [16] selected elderly patients undergoing cancer surgery and obtained the same conclusions, which proved that general anaesthesia by propofol has fewer effects on POCD than sevoflurane inhalation.

However, some other studies showed that there were no differences in the incidence of POCD between propofol anesthesia group and sevoflurane inhalation group [ 17, 18] . Miller *et al*. [19] conducted a meta-analysis and did not obtain a definitive conclusion on the incidence of POCD between propofol and sevoflurane groups. Furthermore, other studies argued that general anesthesia by propofol could induce the cognitive impairment. Previous studies compared the effects of propofol and sevoflurane anesthesia on cognitive function of patients with lung surgery and found that propofol might have more adverse effects on the cognition than sevoflurane [ 20, 21] . Sun *et al*. [22] also conducted a meta-analysis and demonstrated cognitive impairment induced by propofol anesthesia.

The results mentioned above imply whether propofol has an effect on the incidence of POCD remains unclear because uniform standards are lacking for these studies, including the doses of propofol, the effects of other drugs, the severity of surgery, as well as the basic information of patients. In addition, clinical studies mainly focused on the integral role of single general anesthesia and surgery in cognition, rather than the role of anesthesia itself or repeated anesthesia.

## Propofol Anesthesia and POCD in Animal Studies

Many animal studies were performed to explore the effects of propofol anethesia on cognitive function. It has been proven that propofol could attenuate isoflurane/surgery-induced cognitive impairment, cerebral ischemia-reperfusion injury, septic encephalopathy, as well as Alzheimer’s disease (AD) in different disease models [ 23, 24] . The related mechanisms include anti-inflammation, anti-oxidative stress, anti-apoptosis and so on. However, more and more attention has been paid to the adverse effects of propofol with different doses, anesthesia time and periods on the cognition, which may be related to propofol-induced neuro-behavior injuries.

### Age

Aged rodents are the most commonly used animals to explore the link between anesthesia and cognitive function. Several studies reported that propofol could induce cognitive impairment. Chen *et al*. [25] found that 150 mg/kg propofol (intraperitoneal injection, i.p.) could cause cognitive impairment in aged mice, and the damage could last up to 65 days after propofol administration. Yang *et al*. [26] also found that propofol anesthesia administered for 4 h could cause cognitive dysfunction in aged rats. These results proved that in the elderly, propofol anesthesia intervention can impair cognitive function, but the specific effect still needs to be confirmed by further studies.

Similarly, the effect of propofol on infant brain function has been the concern in recent years. Infants and young children are in the developmental stage of neurological function, so any anesthetic factors may affect the functional activities of neurons, synaptic plasticity, *etc*. [ 27, 28] . A large number of basic studies have confirmed that propofol intervention can damage the neurons of newborn rats and cause neuronal apoptosis, which may affect the synaptic structure of the neurons, induce central inflammation, and interfere with the proliferation and differentiation of neural stem cells [29]. Propofol damages the neurons in a dose-dependent manner, and these damages can continue into adulthood [30].

### Dose of anesthesia

In general anesthesia, it is very important to choose the appropriate dose and maintain the appropriate depth of anesthesia. In animal studies, the effects of different doses of propofol anesthesia on cognitive function have also been reported. Anesthetic dose is directly related to anesthetic depth in clinic anesthesia. Woodhouse *et al*. [31] explored the effects of three doses of propofol on cognitive function in AD mice. They found that 50 mg/kg, 100 mg/kg, and 200 mg/kg propofol had no significant effect on the level of β-amyloid (Aβ) in the brain of mice and cognitive function [31]. Zhao *et al*. [32] also explored the effects of propofol with different doses on rat cognition and found that the cognitive ability of rats in the 200 mg/kg group was significantly lower than that of other low-dose groups, suggesting that propofol can damage the cognitive function and that the severity of the damage is dose-dependent. Additionally, with the increase in the dose of propofol, the effect of cognitive impairment became more obvious. However, the discrepancy of these studies may be related to the dosing interval and animal models, and this remains to be clarified.

### Anesthesia time and intervals

In addition to the dosage and depth of anesthesia, the time and intervals of propofol administration also have different effects on cognitive function. Shao *et al*. [33] investigated the effects of propofol with intermittent administration on cognitive function in aged mice with AD. The results suggested that propofol administration with 50 mg/kg once a week could improve the cognitive function of mice, which may be related to the reduction of neuronal apoptosis. Woodhouse *et al*. [31] reported that monthly administration (i.p.) of propofol did not significantly improve the cognitive status of AD mice. Liu *et al*. [34] explored the effects of different dosing intervals on cognitive behavior in aged rats and found that intraperitoneal injection of propofol every 9 days had no significant effect on the spatial learning and memory ability of rats. In contrast, when being administered once a day, propofol could lead to a decrease in memory ability. Thus, it has been suggested that the interval of propofol administration affects cognitive function in aged rats [34]. In addition, the duration of anesthesia also affects cognitive function. Yang *et al*. [26] found that propofol anesthesia administered for 4 h could damage the spatial learning and memory ability, while propofol anesthesia for 2 h did not damage the cognition of aged rats. Zhang *et al*. [25] also found that 50 mg/kg of propofol administration (i.p.) in young mice for 6 h could induce cognitive impairment which could last up to 3 months after the propofol administration. Thus, it has been suggested that prolonged anesthesia increases the risk of cognitive impairment. In clinical practice, propofol infusion syndrome can occur when large doses of propofol are maintained for a long time. Propofol infusion syndrome is a rare clinical complication that manifests as metabolic acidosis, rhabdomyolysis, lipid metabolism disorders, hyperlipidemia, severe arrhythmia, and heart failure [35] ^.^ Therefore, prolonged infusion of propofol should be avoided in clinic.

## Possible Related Mechanisms of the Effects of Propofol on the Cognition

### Inflammation

At present, central inflammation has been considered to be an important factor in the occurrence and development of POCD [36]. Anesthesia and surgery can cause tissue damage and induce the release of pro-inflammatory factors. Inflammatory mediators can damage and infiltrate the blood-brain barrier, activate microglia and astrocytes, and further induce an inflammatory cascade, leading to neuronal damage [37]. Among them, TLR4/MyD88/NF-κB, p38MAPK, PI3K/Akt, and other signaling pathways are activated in the regulation of inflammation [ 38– 40] . According to the effects of propofol on cognition, many studies have reported that propofol intervention can effectively inhibit central inflammation caused by surgery, hypoxia, as well as infection, and exert a cognitive protective effect [41]. The anti-inflammatory effects of propofol have been confirmed in various animal models [42]. However, some studies have found that treatment with a certain concentration and dose of propofol can cause non-specific inflammation, which in turn leads to central inflammation and impaired cognitive function. Liu *et al*. [34] found that repeated propofol injections (200 mg/kg) could induce peripheral and central inflammation, activate the NF-κB signaling pathway and NLRP3 inflammasome, and cause neuronal damage [34]. Zhu *et al*. [42] also found that propofol leads to an increase in the levels of serum inflammatory factors, such as interleukin-1β and tumor necrosis factor-α (TNF-α), and a decrease in interleukin-10 level, which in turn impairs cognitive function [42]. Some previous studies also proved that inflammatory factors were significantly increased after surgery under propofol anesthesia. Zhang *et al*. [43] also found that propofol could activate the NF-κB signaling pathway in young mice, and induce inflammation and cognitive dysfunction.

### Abnormal function of related proteins

Previous studies have confirmed that Aβ and tau protein are related to the early onset of AD and are closely related to neurological damage [44]. Similarly, Aβ and tau protein were both confirmed to be involved in the occurrence of POCD [45]. However, the effect of propofol on the expressions of Aβ and tau protein remains controversial. Berger *et al*. [46] found that surgery could lead to an increase of Aβ and tau in the cerebrospinal fluid of patients, but there was no clear correlation with anesthetics. Woodhouse *et al*. [31] also showed that propofol had no effect on central Aβ protein expression in a mouse model of AD. However, some other studies found that propofol could promote the expression of Aβ protein and phosphorylation of tau protein. Zheng *et al*. [47] found that single propofol injection could promote the phosphorylation of tau protein, rather than the amyloid precursor protein, in the hippocampus of aged rats and induce cognitive impairment. Furthermore, Zou *et al*. [48] found that different sedation depths of propofol affect the levels of Aβ(1–42) and p-tau in the cerebrospinal fluid. In patients with relatively deep sedation, Aβ(1–42) and p-tau levels in the cerebrospinal fluid were significantly increased, suggesting a connection to cognitive impairment. Huang *et al*. [49] found that propofol anethesia for 2 h greatly increased the phosphorylation of tau in the hippocampus and cortex of adult rats, by promoting the level of protein phosphatase 2A (PP2A), which is the major tau phosphatase. In addition, Chen *et al*. [25] proved that propofol could promote the phosphorylation of tau, while insulin inhibited the effect of propofol by reducing the level of PP2A in the brain. However, due to the influence of different animal models and different intervention methods, the effect of propofol on Aβ and tau protein phosphorylation remains controversial, which needs to be clarified in the future.

Previous studies have also reported the role of α-synuclein in the cognitive impairment associated with propofol. α-Synuclein is mainly present in the presynaptic terminals. It may participate in the maintenance of neurotransmitter homeostasis by regulating the fusion of synaptic vesicles and binding with neurotransmitter membrane transporters [50]. α-Synuclein is the non-Aβ component of amyloid plaques in the brains of AD patients [51]. Yang *et al*. [26] revealed for the first time that propofol anesthesia could up-regulate the level of central α-synuclein, leading to neurotransmitter disorders and cognitive impairment, which provided a new direction for subsequent research.

### Serious imbalance of neurotransmitter levels

The balance of neurotransmitters is vital for the cognitive function of the brain. Acetylcholine is one of the most studied neurotransmitters, which is widely distributed in the brain. Acetylcholine is involved in brain memory function, attention, sleep, and the regulation of neuronal activity in the hippocampus and neocortex [52]. Among them, M-cholinergic receptors are the basis for human memory function and affect the establishment of nerve synapses. In clinic, anesthetics such as propofol have an inhibitory effect on acetylcholine receptors, which can cause cognitive impairment [53]. Acetylcholine, glutamate receptors (Glu), and γ-aminobutyric acid receptors (GABA) are also involved in the process of cognitive activities and synaptic plasticity [54]. Glu receptors are excitatory receptors that increase the long-term potential of synapses by promoting the flow of calcium ions into neurons, and improve synaptic plasticity and neuronal activity [55]. Therefore, changes in Glu/GABA ratio have been a hot research topic in recent years. Tu *et al*. [56] found that propofol treatment downregulated the ratio of Glu/GABA, which in turn impaired synaptic plasticity and neuronal function, leading to cognitive impairment. Wu *et al*. [57] also found that propofol could reduce Glu level and affect the memory in young rats. *In vitro* experiments also confirmed that propofol inhibited the phosphorylation of Glu in the hippocampus, leading to the obstruction of long-term potential formation [58].

### Abnormal synaptic plasticity

Synaptic plasticity is the basis for learning, which regulates cognitive function. Therefore, inhibition of synaptic plasticity, including long-term potentiation (LTP) and long-term inhibition, is closely related to neurodegenerative diseases [59]. Synaptic plasticity is closely related to neurotransmitters. Glutamate is considered to be the basis for memory and an important excitatory neurotransmitter in the brain, which plays a special key role in the formation of LTP. GABA neurotransmitters inhibit long-term enhancement, leading to impairment of learning and memory [60]. Many previous studies have explored the effects of propofol on long-term potentials and revealed its role in cognitive impairment [61]. Gao *et al*. [62] found that repeated propofol injections in young rats could inhibit long-term potentials and impair spatial learning and memory. Peng *et al*. [63] also confirmed that propofol could inhibit hippocampal neurodevelopment and lead to cognitive impairment. Zhong *et al*. [64] found that the inhibitory effect of propofol on long-term potentials in young mice was related to the dose, but there was no clear correlation. Unfortunately, there are relatively few reports on aging studies. Li *et al*. [65] reported that propofol inhibits long-term potentials in the elderly, which is more severe than those in the adults. These results imply that aging may affect the inhibitory effect of propofol on LTP. It has been suggested that the effect of propofol on LTP is also related to aging.

### Abnormal autophagy

Autophagy is a lysosomal-dependent degradation pathway involved in the regulation of cell survival, proliferation and differentiation. Autophagy maintains normal functions of cells and organelles by degrading abnormally accumulated proteins or damaged organelles [66]. In neurons, autophagy could clear the Aβ protein and phosphorylated tau protein in the brain. When autophagy function is impaired, the clearing ability decreases, leading to cognitive dysfunction [67]. In recent years, it has been reported that the decrease of autophagy level is closely related to the occurrence of neurodegenerative diseases [68]. Some studies have also reported the effect of propofol on autophagy [69]. Yang *et al*. [26] found that propofol anesthesia could inhibit the level of autophagy, promote abnormal accumulation, and induce cognitive impairment. In addition, propofol could promote insulin secretion, activate PI3K signaling pathway through insulin receptor, and have biological functions [70]. Some other studies suggested that propofol could also inhibit the over-activation of autophagy induced by ischemia and hypoxia, thereby playing a neuro-protective role [71]. However, the specific regulatory mechanisms of propofol and autophagy are still controversial and need to be explored in further studies.

### Non-coding RNA regulation

MiRNAs are small non-coding RNAs that exert their biological functions by regulating post-transcriptional gene expression. In recent years, studies have found that miRNAs play an important role in brain development, neuronal differentiation, synaptic connections, and dendritic spine formation [72]. Bioinformatics analysis of changes in the expressions of miRNAs in the hippocampus of POCD mice revealed that 22 miRNAs were differentially expressed. These miRNAs may be related to the pathogenesis of POCD, and they have attracted increasing interests in recent years [73]. Subsequent studies reported the role of miRNAs in propofol anesthesia. In young mouse models, Sun *et al*. [74] found that propofol anesthesia can up-regulate the expression of miR-183, thereby inhibiting the differentiation of neural stem cells and other biological activities, affecting learning and memory. In addition, previous studies have found that propofol can also up-regulate miR-206 [75]. Neuronal apoptosis or inhibition of miR-132 affects learning and memory [76]. Meanwhile, some studies reported that histone modification and DNA methylation may be related to propofol-induced cognitive injury. Holtkamp *et al*. [77] found that propofol could inhibit the activity of AChE/BChE in an *in vitro* model by reducing the methylation of *CHRNA7* and *H3K27* gene. Zhang *et al*. [78] also found that repeated propofol anesthesia could promote the CpG site hypermethylation of the *EFEMP1* gene, which may be involved in the cognitive impairment. However, the specific link between DNA methylation and propofol remains to be clarified by further studies.

## Conclusions and Perspectives

**Figure 1:**
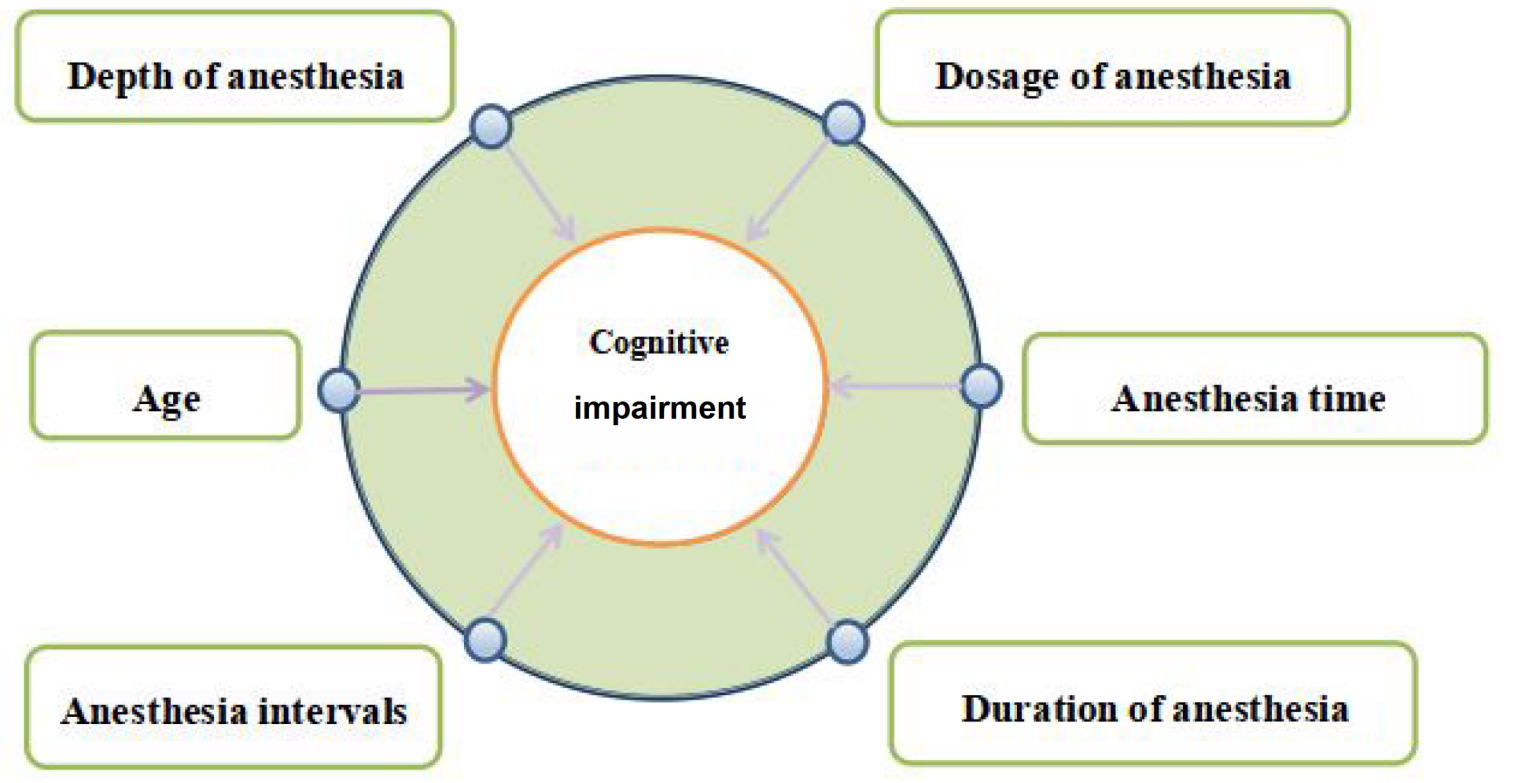
Figure 1 Related factors on propofol-induced cognitive impairment Propofol-induced cognitive impairment is associated with several factors, such as age, the dose of propofol, anesthesia depth, anesthesia time, anesthesia duration, as well as anesthesia intervals.

In summary, whether propofol plays a protective or adverse role in cognitive function remains in dispute. The results of clinical studies just showed the effects of propofol anesthesia combined with surgery on the cognition, rather than a single anesthetic or surgical factor. Thus, based on the basic and clinical studies, we believe that some factors, such as age, the dose of propofol, anesthesia depth, the time and intervals of propofol anesthesia may be involved in the regulation of cognition by propofol ( Figure 1).

**Figure 2:**
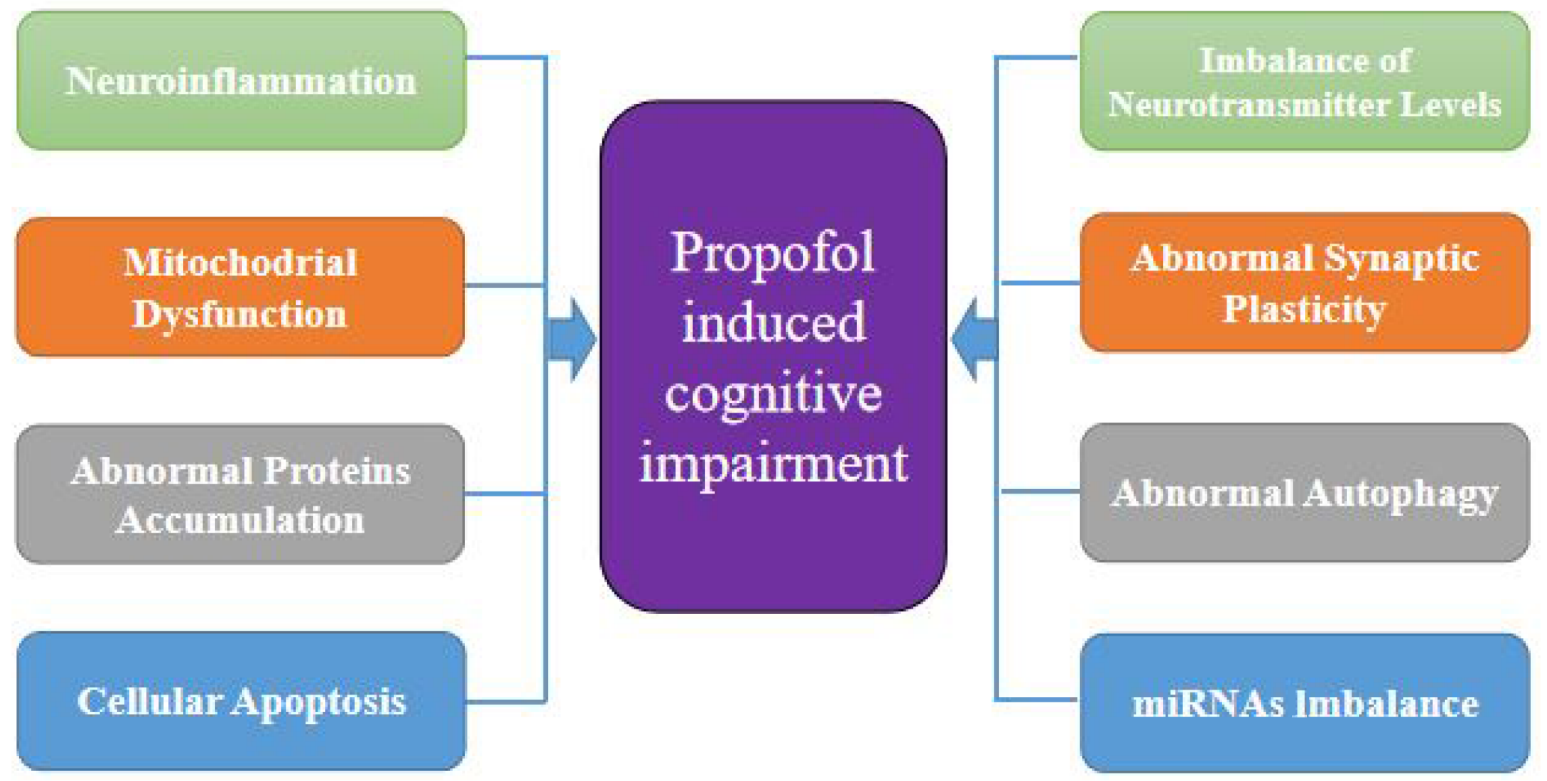
Figure 2 Potential mechanisms of propofol-induced cognitive impairment Excessive propofol anesthesia could induce neuro-inflammaiton, mitochondrial dysfunction, abnormal protein accumulation (including tau protein, α-synuclein, etc.), cellular apoptosis, imbalance of neurotransmitter level (abnormal Glu/GABA ratio), impaired synaptic plasticity, abnormal changes in autophagy, as well as abnormal expressions of miRNAs.

In infants, young children, and the elderly, propofol may induce a decline in learning and memory. Propofol anesthesia with high dose can also promote the occurrence of cognitive impairment. In addition, long-term propofol injections or repeated continuous injections of propofol may impair cognitive function. The specific influencing factors are mainly related to inflammation, abnormal proteins accumulation, abnormal neurotransmitter balance, impaired synaptic plasticity, abnormal levels of autophagy and related miRNA molecules ( Figure 2). Nevertheless, more studies are needed to reveal the regulatory mechanism of propofol’s influence on cognition and the interaction of various factors.
